# Redescription of *Gammarus pseudosyriacus* (Karaman & Pinkster, 1977) and description of a new subspecies from southern Iran (Crustacea, Amphipoda, Gammaridae)

**DOI:** 10.3897/zookeys.598.8064

**Published:** 2016-06-14

**Authors:** Maryam Semsar-Kazerooni, Mehrdad Zamanpoore, Saber Sadeghi

**Affiliations:** 1 Department of Biology, Faculty of Sciences, Shiraz University, Adabiiat Crossroad, Shiraz, Fars, Iran; 2 Department of Hydrobiology and Fisheries, Agricultural Research, Education, and Extension Organization, Modarres Boulevard, Shiraz, Fars, Iran

**Keywords:** Taxonomy, amphipod, Zagros, Yazd, Fars, invertebrate, freshwater, Gammarus pseudosyriacus

## Abstract

The present study focused on redescription of *Gammarus pseudosyriacus* (Karaman & Pinkster, 1977) based on new materials from Zagros Mountains and describes a new subspecies of freshwater amphipod, *Gammarus pseudosyriacus issatisi* **subsp. n.**, from the southern Zagros Mountains. The work is based on morphological and morphometric comparisons. This new subspecies has features similar to *Gammarus pseudosyriacus*. The distinct features that distinguish *Gammarus pseudosyriacus issatisi* subsp. n. from *Gammarus pseudosyriacus* are the smaller eyes, shorter body length, and shorter flagellum of antenna 1 and 2.

## Introduction

*Gammarus* Fabricius, 1775 is the largest genus among the amphipod genera and is widespread throughout the northern hemisphere (Karaman and Pinkster 1977). By 2008 more than 200 species of *Gammarus*, which have the highest diversity in Palearctic region, especially in the Mediterranean mountains and Near East, had been described (Väinölä et al. 2008). Until now 18 species of *Gammarus* have been reported from the freshwater regions of Iran (Zamanpoore et al. 2011).

*Gammarus pseudosyriacus* Karaman & Pinkster, 1977 is distributed in Syria (surroundings areas of Damascus), Turkey, Afghanistan (Karaman and Pinkster 1977). This species is also distributed in all parts of the Zagros Mountains in Iran: northern, central and southern Zagros (Zamanpoore et al. 2011). This species has a wide tolerance range to temperature (usually 5–21 °C), so it seems that this adaptation to different water temperatures is the main reason for its wide distribution (Zamanpoore et al. 2011).

*Gammarus pseudosyriacus* was described in Karaman and Pinkster (1977); however, because of the high number of new species described in their publication, all descriptions including that of *Gammarus pseudosyriacus* are minimal. Likewise, few illustrations of body parts were provided. This may cause problems in identification, especially in the case of *Gammarus pseudosyriacus* due to its wide range of distribution and hence the probability of high morphological variation which requires detailed descriptions. Therefore, a redescription of *Gammarus pseudosyriacus* based on new materials is given here. Due to the various catchment basins in the southern Zagros region, many isolated populations of aquatic organisms exist, and consequently, the probability of forming new subspecies and species is high (Zamanpoore et al. 2010). This paper presents results of the investigation of samples of two endemic populations from springs, one in Fars province, and the second from Yazd province, Iran. Each spring is surrounded and separated by desert plains and these plains provide geographical barriers between the two populations.

The aim of this study is to prepare a redescription of *Gammarus pseudosyriacus* based on materials in its more central distribution range inside the Zagros Mountains, and to describe a new subspecies from a population in the adjacent marginal range.

## Methods

Specimens were collected by hand nets. Washed and cleaned specimens were preserved in 70% ethanol in the field. Thirty adult male specimens of each population were stained with Lignin Pink, dissected under a stereomicroscope (Zeiss, Stemi SV11), and dissected parts were mounted on a temporary slide with glycerine for examination under a compound microscope (Zeiss, Stemi IV6). Digital microphotographs of body parts were taken by a digital camera (Oculer, 3MPCCD). These photos were used for measuring all body parts of two populations with IMAGE TOOL software (V.3.0, 2002, UTHSCSA) and also to make digital drawings in CORELDRAW (V.11.633, 2002, Corel Corporation). By using the word spine in all parts of this paper, we mean “spinniform setae” as defined by Oshel and Steele (1988).

All specimens are stored in the amphipod collection of the Museum of Fars Research Centre of Agriculture and Natural Resources Aquatic Invertebrate Collection (FAIC), Shiraz, Iran and in the Zoological Museum of Shiraz University, Collection of Biology Department, Shiraz, Iran (ZM–CBSU).

Some environmental factors that were measured in both locations include salinity, pH, electrical conductivity, water temperature and water depth.

## Taxonomy

### Gammarus pseudosyriacus pseudosyriacus

Taxon classificationAnimaliaAmphipodaGammaridae

Karaman & Pinkster, 1977

Gammarus pseudosyriacus Karaman & Pinkster, 1977: 55–58, fig. 22

#### Type locality.

**Figure 1.**
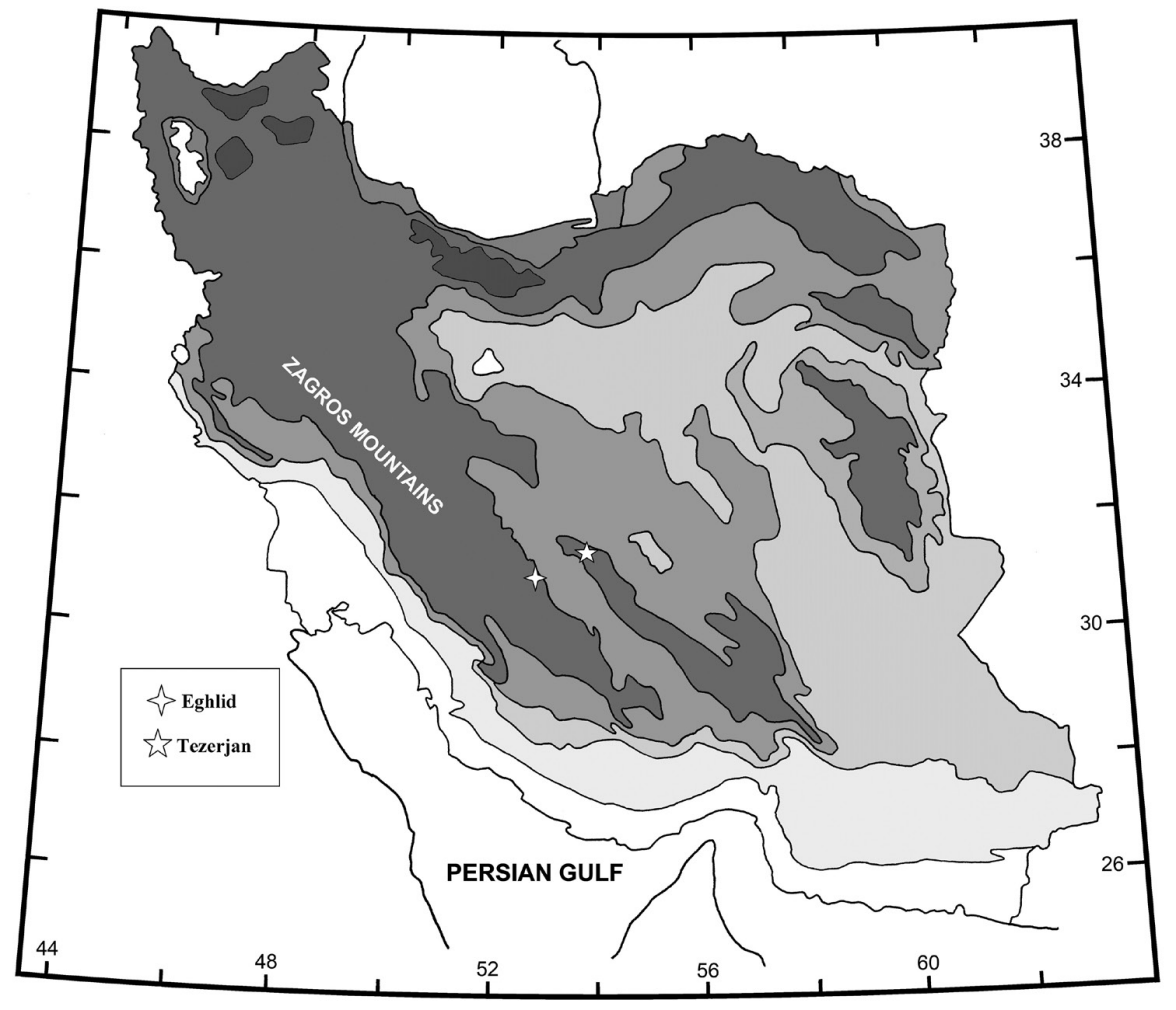
Map of sampling sites, Eghlid and Yazd stations.

The type locality of *Gammarus pseudosyriacus* Karaman & Pinkster, 1977 is small pools in surroundings of Damascus. The samples were collected from springs and qanats of Zagros Mountains in October 2012. Location was Eghlid station (Rasoul Spring, Eghlid, Fars province, Iran, 30°53'27.6"N; 52°40'18.3"E, Altitude 2167 m) (Fig. 1). Leg. M. Semsar-Kazerooni.

#### Material examined.

The description is based on new material collected by the authors from Eghlid, Fars, Iran, a locality inside the distribution range of the species (Turkey, Iran, Afghanistan, Israel and Syria). Eight males were completely dissected and examined in detail, and compared to another 22 males (FAIC 111300, ZM–CBSU #3210). One male, with genitalia in a separate microvial. Original label: “‌FAIC 111300, Eghlid, Rasoul Spring, 30°53'27.6"N; 52°40'18.3"E, 15 October 2012”. As well as to samples from previously collected material from winter, spring, and summer.

#### Description.

**Figure 2.**
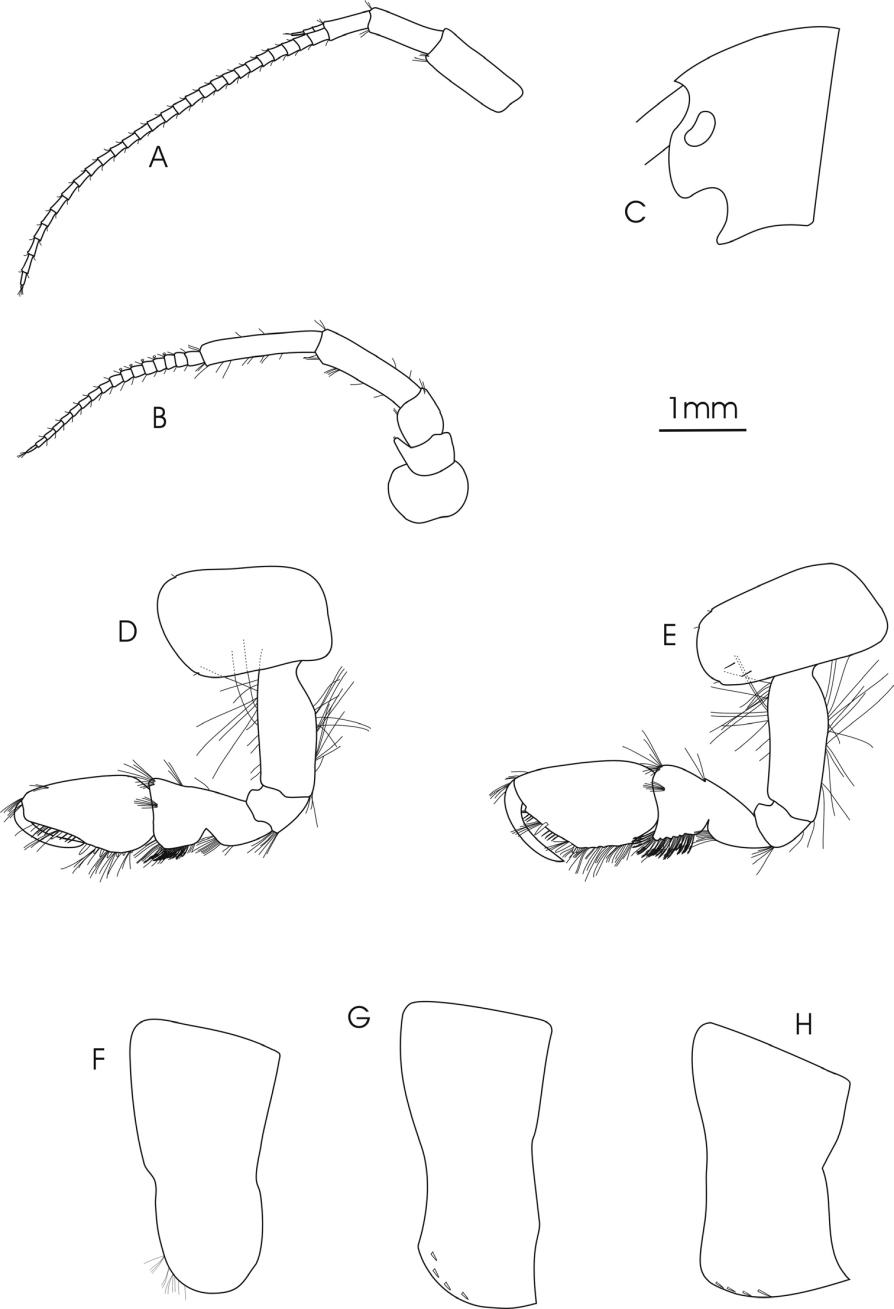
*Gammarus pseudosyriacus pseudosyriacus*, ♂, 20 mm. **A** antenna 1 **B** antenna 2 **C** head **D** gnathopod 1 **E** gnathopod 2 **F–H** epimeral plates1–3.

**Figure 3.**
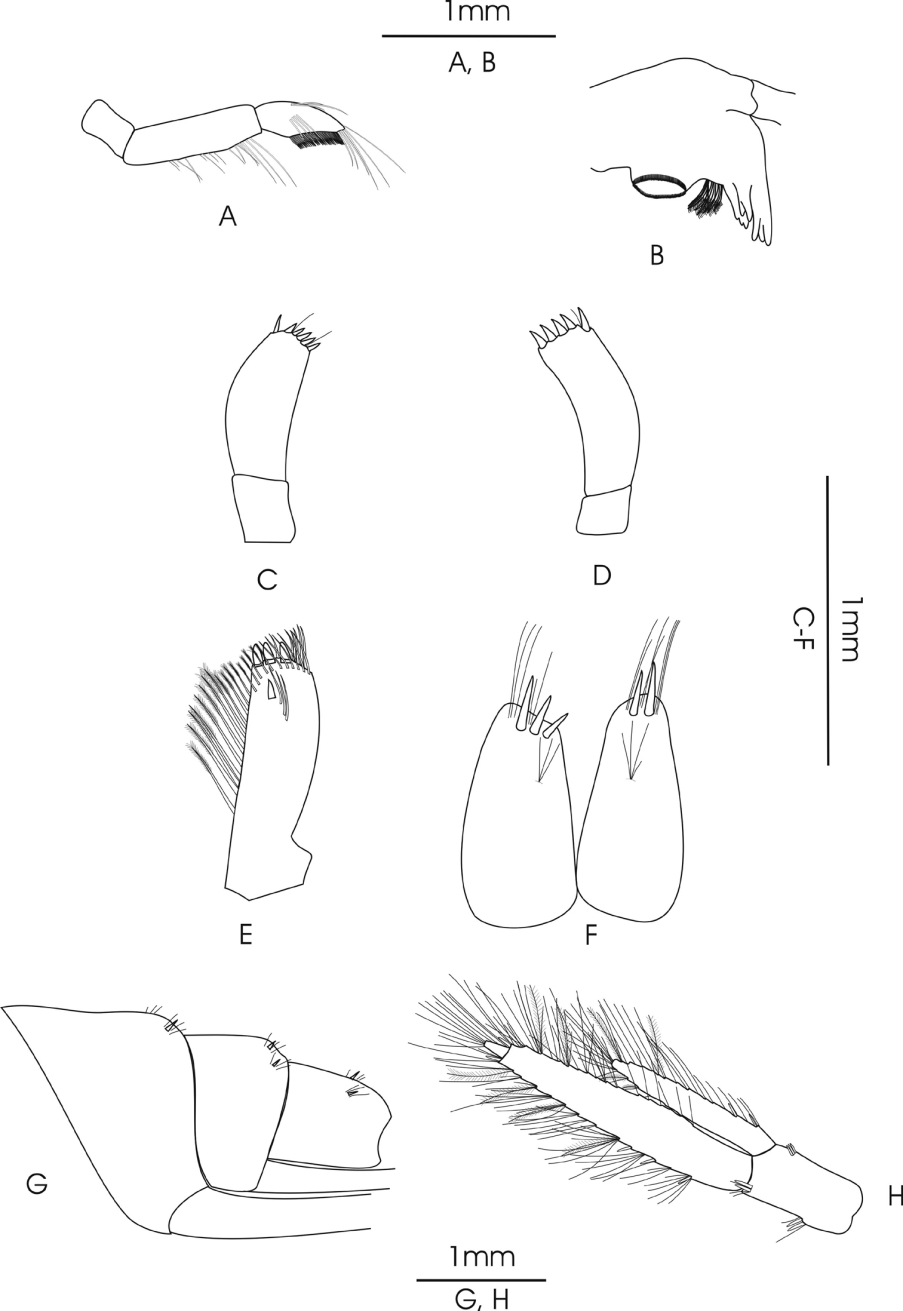
*Gammarus pseudosyriacus pseudosyriacus*, ♂, 20 mm. **A** mandible palp **B** mandible **C** palp of left maxilla **D** palp of right maxilla **E** exopodite of maxilliped **F** telson **G** urosomites **H** uropod 3.

Maximum body length 22 mm; kidney-shaped and medium-sized eyes (the length of which are equal to the diameter of the first peduncular article of antenna 1) (Fig. 2C); sharp epimeres (Fig. 2F–H) and clearly elevated urosome segment (Fig. 3G). *Antenna 1*: Longer than antenna 2; peduncular articles 1>2>3; main and accessory flagella with 22–35 and 2–5 articles, armed with short simple setae (Fig. 2A). *Antenna 2*: Gland cone is shorter than the third peduncle article; peduncle articles 4 and 5 about equal length and armed with groups of short setae; flagellum with 10–18 articles and also armed with short simple setae; calceoli present (Fig. 2B). *Mandible*: All parts include incisor processes, *lacinia mobilis* and ridged molar process well developed, also a plumose long spine row is present (Fig. 3B). *Mandible palp*: First article without setae; second article with ventral setae, 3–6 proximal setae and 9–13 closely placed distal setae; inferior margin of the third article armed with a comb-like row of 30–36 D-setae, 5–6 long E-setae, one group of B-setae and one group of A-setae (Fig. 3A). *Maxilla 1*: Inner basal lobe with plumose setae; stout serrate spines on outer lobe; palps asymmetric; right palp with 4 robust tooth-like spines on apical margin, one longer separate subapical spine on its outer margin with one seta (Fig. 3D). Left palp with 5 apical spines accompanied by 2 median setae, one longer separate subapical spine on outer corner (Fig. 3C). *Maxillipeds*: Exopodite with a row of 3 strong teeth and 6 longer setae on distal margin, a row of setae at distal sub-margin which becomes plumose from the middle and continues towards the inferior margin to join to 8–10 long plumose setae, a single spine with a distance at sub-marginal interior corner, a row of three setae parallel to the long axis close to the single spine (Fig. 3E).

**Figure 4.**
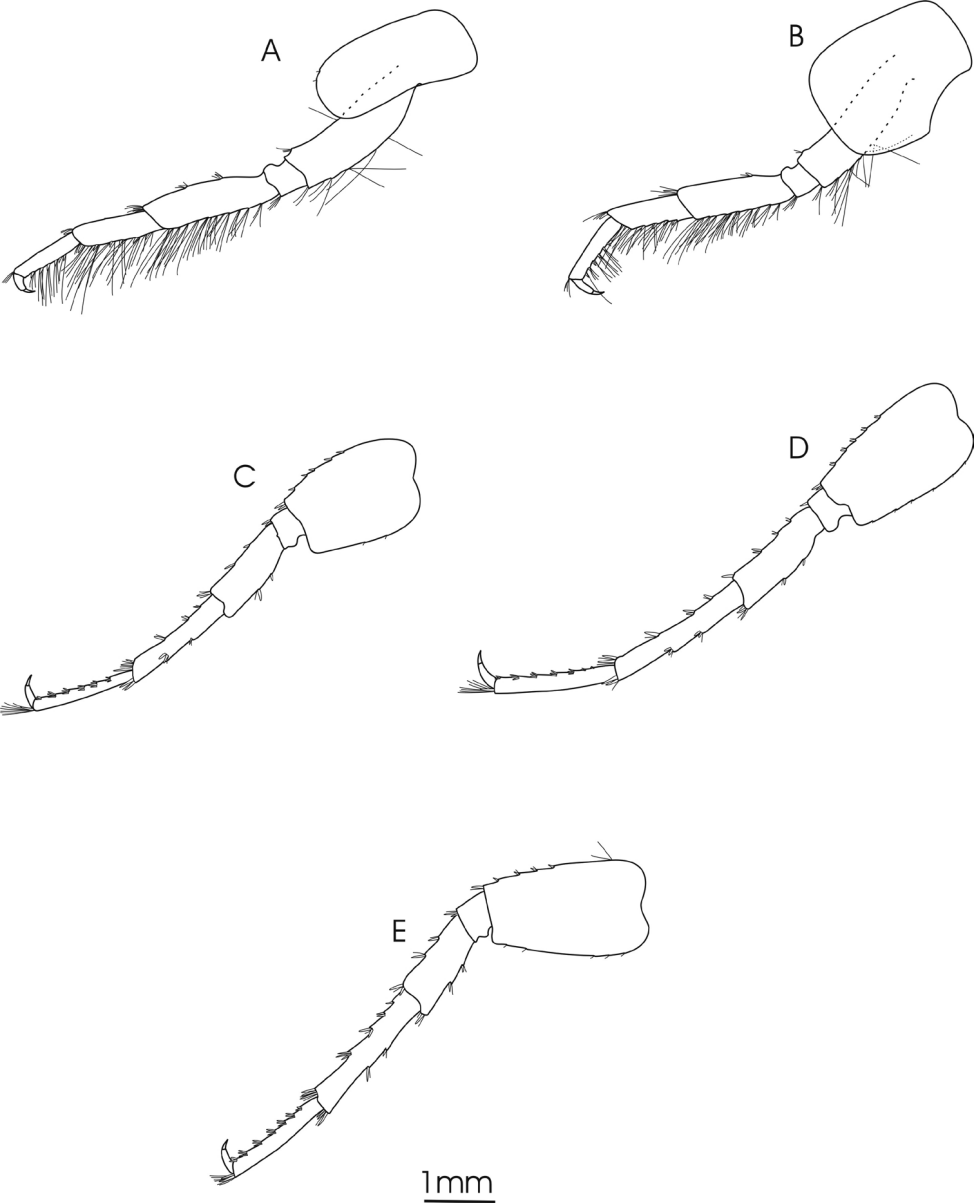
*Gammarus pseudosyriacus pseudosyriacus*, ♂, 20 mm. **A** pereopod 3 **B** pereopod 4 **C** pereopod 5 **D** pereopod 6 **E** pereopod 7.

*Gnathopod 1*: Coxal plate distally slightly wider than proximal, rounded corners with a seta at the postero-ventral corner and 1–2 setae at antero-ventral corner; basis with a few long setae on both anterior and posterior margins; ischium with a postero-distal row of setae; merus and carpus with groups of short setae which are plumose at posterior margin of carpus; propodus pyriform with groups of spines and setae, 6–7 groups of small spines at posterior palmar margin; dactylus long (Fig. 2D). *Gnathopod 2*: Coxal plate distally slightly narrower than proximal, rounded corners with a seta at the postero-ventral corner and 1–3 setae at antero-ventral corner; basis with a few long setae on both anterior and posterior margins; ischium with a postero-distal row of setae; merus and carpus with groups of short setae which are plumose at posterior margin of carpus; propodus trapezoid-shaped (subrectangular) with 3 groups of spines, and also groups of dense setae on palmar surface (Fig. 2E). *Pereopod 3*: Coxal plate rectangular and rounded distally, with two short setae at antero-distal corner and one at postero-distal corner; anterior and posterior margins of basis bear some long simple setae; posterior margins of merus and carpus densely setose; merus with several groups of dense setae on posterior margin about 1 to 1.5 times as long as the diameter of the article, anterior margin of merus with 2 groups of short spine that intermixed with short setae and a group of long setae and a spine at anterior tip; posterior margin of carpus with several groups of dense setae 2 times longer than the diameter of the article, a long spine and a group of longer setae are implanted on both its anterior and posterior tip; posterior margin of propodus with 6–7 groups of small spines and some long setae (Fig. 4A). *Pereopod 4*: Coxal plate with a small seta implanted at antero-distal margin and 1–4 at postero-distal margin; articles similar to pereopod 3, but setae are shorter and the number of setae is lower; anterior margin of merus with just one group of short setae and spines, long spines implanted at anterior tip among a group of setae; posterior margin of carpus with several groups of setae and spines; posterior margin of propodus with 6–8 groups of small spines and some long setae (Fig. 4B). *Pereopod 5*: Basis subrectangular, postero-distal lobe well developed, posterior margin with 2–5 very short setae, anterior margin with 4–6 spines mixed with a fine seta; merus and carpus with small spines and setae; propodus having 6–7 transverse rows of spines (Fig. 4C). *Pereopod 6*: Longer than pereopod 5; basis slender and posterior margin with 6–10 setae and anterior margin with 4–6 spines; other articles are similar to pereopod 5 (Fig. 4D). *Pereopod 7*: Basis wider proximally, postero-distal protruding lobe less developed than pereopod 6, posterior margin with 5–11 setae and anterior margin with 4–6 spines; anterior margin of merus and carpus with spines and longer setae; merus with two spines mixed with short setae at posterior margin; carpus with 2–3 spines at posterior margin; propodus with spines and setae which are as long as spines, 6–7 transverse rows of spines on anterior margin of propodus, two longer spines at posterior tip of propodus intermixed with a group of longer setae (Fig. 4E). *Uropod 3*: Endopodite length is about two-thirds of the exopodite; setae on outer and inner margin of both exopodite and endopodite are plumose (Fig. 3H).

*Telson*: Length of the lobes about twice their widest width; 2–5 long and robust spines and 5–8 long setae on distal margin; groups of setae at the dorsal surface of the lobes (Fig. 3F). *Epimeral plate 1*: Rounded with 12–14 long setae on antero-distal corner (Fig. 2F). *Epimeral plate 2*: Posterodistal corner pointed; distal margin with 2–7 short spines (Fig. 2G). *Epimeral plate 3*: Posterodistal corner sharply pointed; 3–5 short spines intermixed with short setae on distal margin (Fig. 2H). *Urosomites 1–3*: With clear dorsal elevation; each urosomite bears a dorsomedian and dorsolateral groups of short setae mixed with short spines on their posterior margins (Fig. 3G).

#### Distribution.

The species is dispersed from Israel to Syria, Turkey, Iran and Afghanistan (Karaman and Pinkster 1977, Zamanpoore et al. 2011). In Iran, it is widespread in the inner parts of the Zagros Mountains, extending from northwest to southeast.

#### Ecology.

Rasoul Spring is covered by a gravel bed and some submersed aquatic plants. Ecological factors include salinity (0.19 g/lit), pH (6.5), electrical conductivity (350 μS/cm), water temperature (15 to17 °C), and water depth (25 cm).

### Gammarus pseudosyriacus issatisi subsp. n.

Taxon classificationAnimaliaAmphipodaGammaridae

http://zoobank.org/9353F82E-30E5-4657-90C9-96A17AABF7C4

#### Type locality.

The samples were collected from springs and qanats of Zagros Mountains in May 2013. Location was in Yazd station (Qanat-e-Hojjat Abad, Tezarjan, Yazd province, Iran, 31°36'20.9"N; 54°10'43.4"E, Altitude 2162 m) (Fig. 1). leg. M. Semsar-Kazerooni.

#### Material examined.

Holotype male, 16.9 mm, Qanat-e-Hojjat Abad, Yazd, Iran, many paratypes, eight males were completely dissected and examined in detail, and compared to another 22 males (FAIC 111299, ZM–CBSU #3209).

#### Type specimen.

Holotype male, with genitalia in a separate microvial. Original label: “‌FAIC 111299, Yazd, Tezerjan Qanat, 31°36'20.9"N; 54°10'43.4"E, 12 May 2013”.

#### Diagnosis.

Small body (maximum length 17 mm), small eyes (smaller than diameter of first peduncular article of antenna 1) with a wider appearance, shorter flagellum of antenna 1 and 2, wider uropod 3, wider telson, wider merus in pereopods 3–6, wider carpus in third, fifth and sixth pereopods, wider basis in pereopod 4, longer basis in pereopod 6 and pereopod 7.

#### Description.

**Figure 5.**
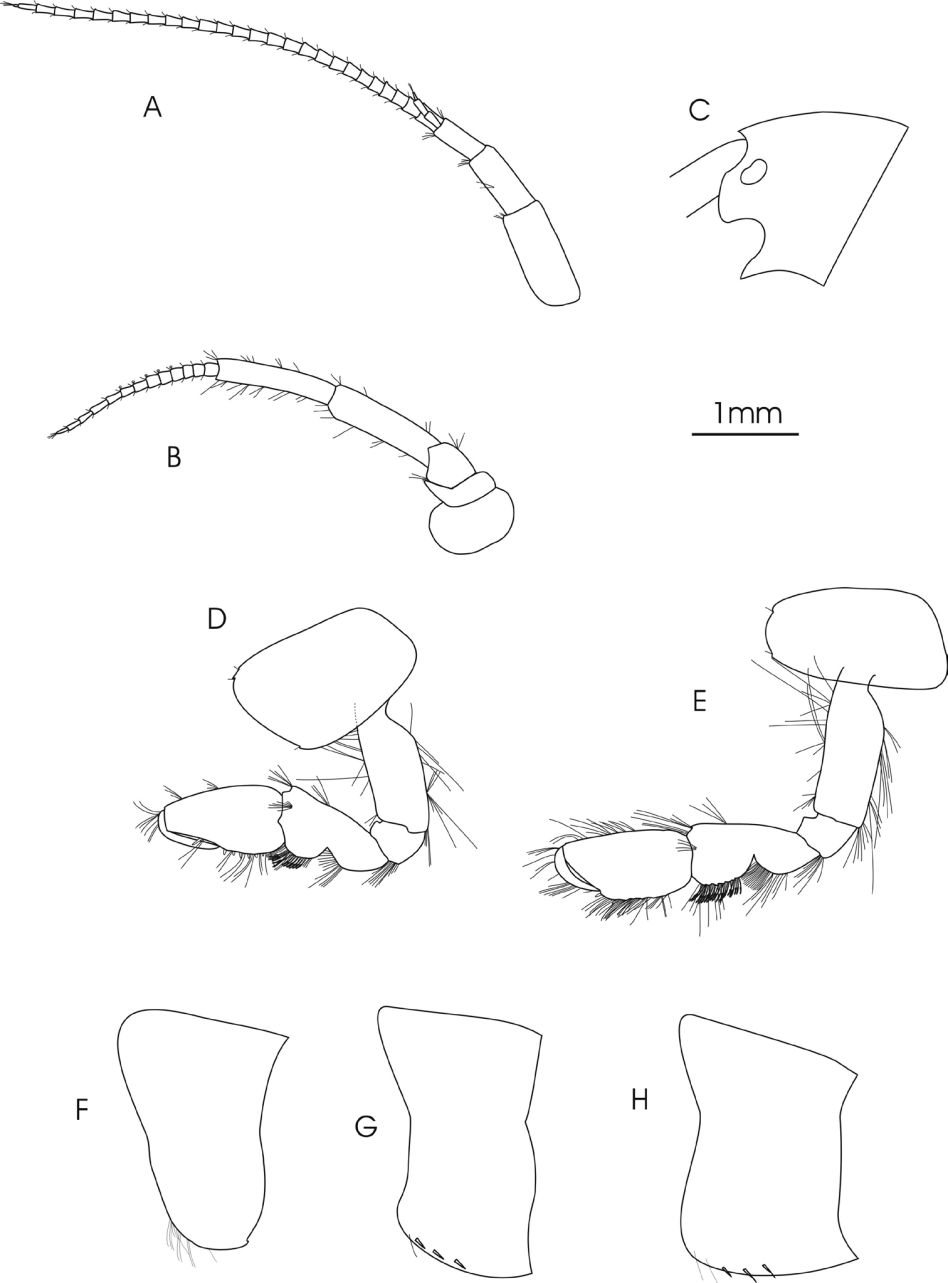
*Gammarus pseudosyriacus issatisi* subsp. n., ♂, 16.9 mm. **A** antenna 1 **B** antenna 2 **C** head **D** gnathopod 1 **E** gnathopod 2 **F–H** epimeral plates 1–3.

**Figure 6.**
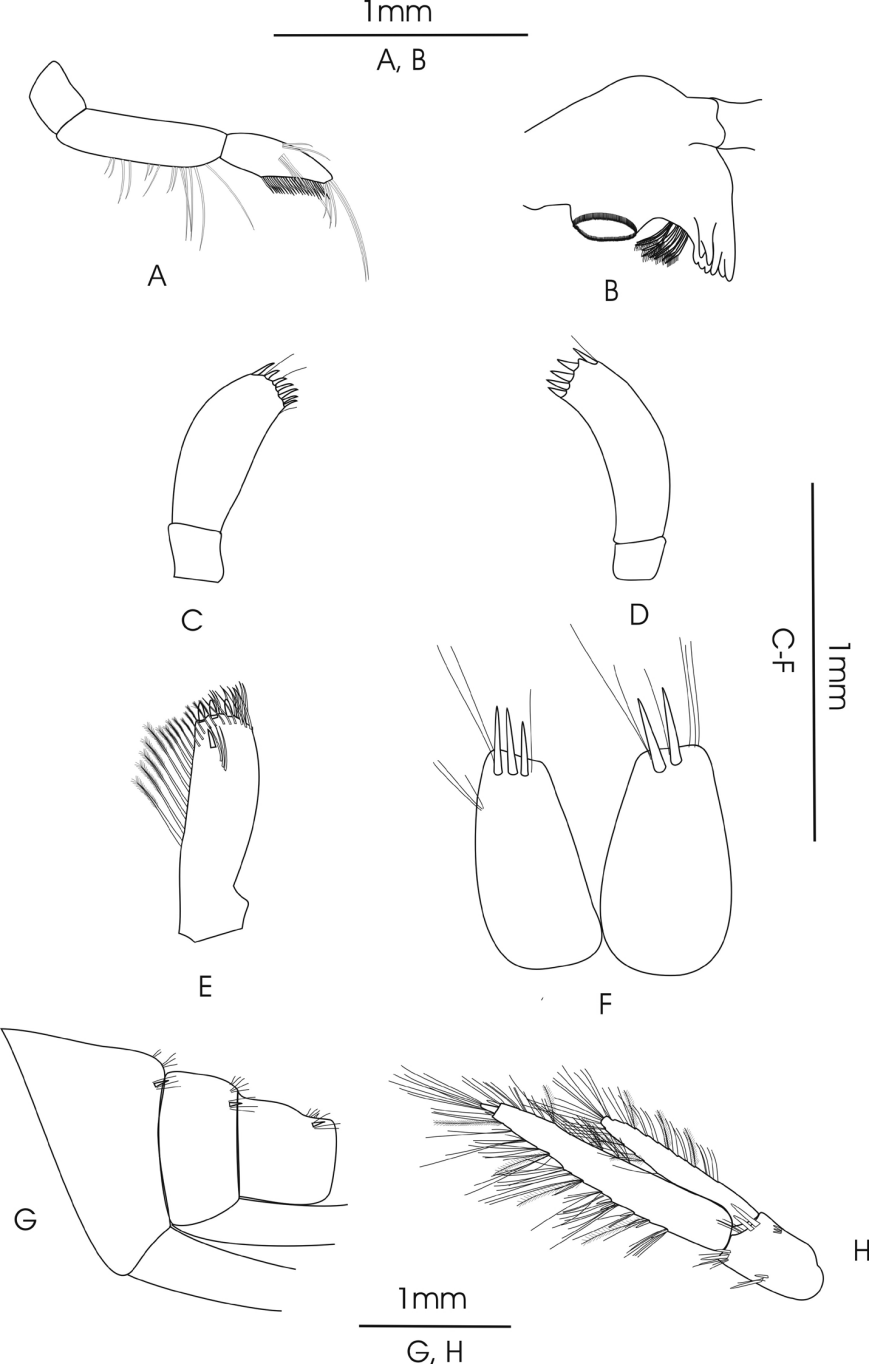
*Gammarus pseudosyriacus issatisi* subsp. n., ♂, 16.9 mm. **A** mandible palp **B** mandible **C** palp of left maxilla **D** palp of right maxilla **E** exopodite of maxilliped **F** telson **G** urosomites **H** uropod 3.

Maximum body length 17 mm; small, kidney-shaped eyes (smaller than diameter of first peduncular article of antenna 1) (Fig. 5C). *Antenna 1*: Longer than antenna 2; peduncular articles 1>2>3; main and accessory flagella with 17–31 and 3–5 articles, armed with short simple setae (Fig. 5A). *Antenna 2*: Gland cone is shorter than the third peduncle article; peduncle articles 4 and 5 approximately equal length and armed with groups of short setae; flagellum with 11–15 articles armed with short simple setae; calceoli present (Fig. 5B). *Mandible*: Incisor processes, *lacinia mobilis* and ridged molar process well developed, a plumose long spine row exist (Fig. 6B). *Mandible palp*: First article without setae; second article with ventral setae, 4–5 proximal setae and 6–11 closely placed distal setae; inferior margin of the third article armed with a comb-like row of 20–26 D-setae, 5–6 long E-setae, a groupof B-setae and a group of A-setae (Fig. 6A). *Maxilla 1*: Long plumose setae on inner lobe; outer lobe with stout serrate spines; palps asymmetric; right palp with 4 robust tooth-like spines, one longer separate subapical spine with one long seta on its outer margin (Fig. 6D). Left palp with 6 apical spines accompanied by 2 median setae and a long subapical seta on inner corner, one longer separate subapical spine on outer corner (Fig. 6C). *Maxillipeds*: Distal margin of exopodite with a row of three strong teeth and 8 longer setae, a row of setae at distal sub-margin which becomes plumose from the middle and continues towards the inferior margin to join to 6–7 long plumose setae, a single spine with a distance at sub-marginal interior corner, a row of three setae parallel to the long axis close to the single spine (Fig. 6E).

**Figure 7.**
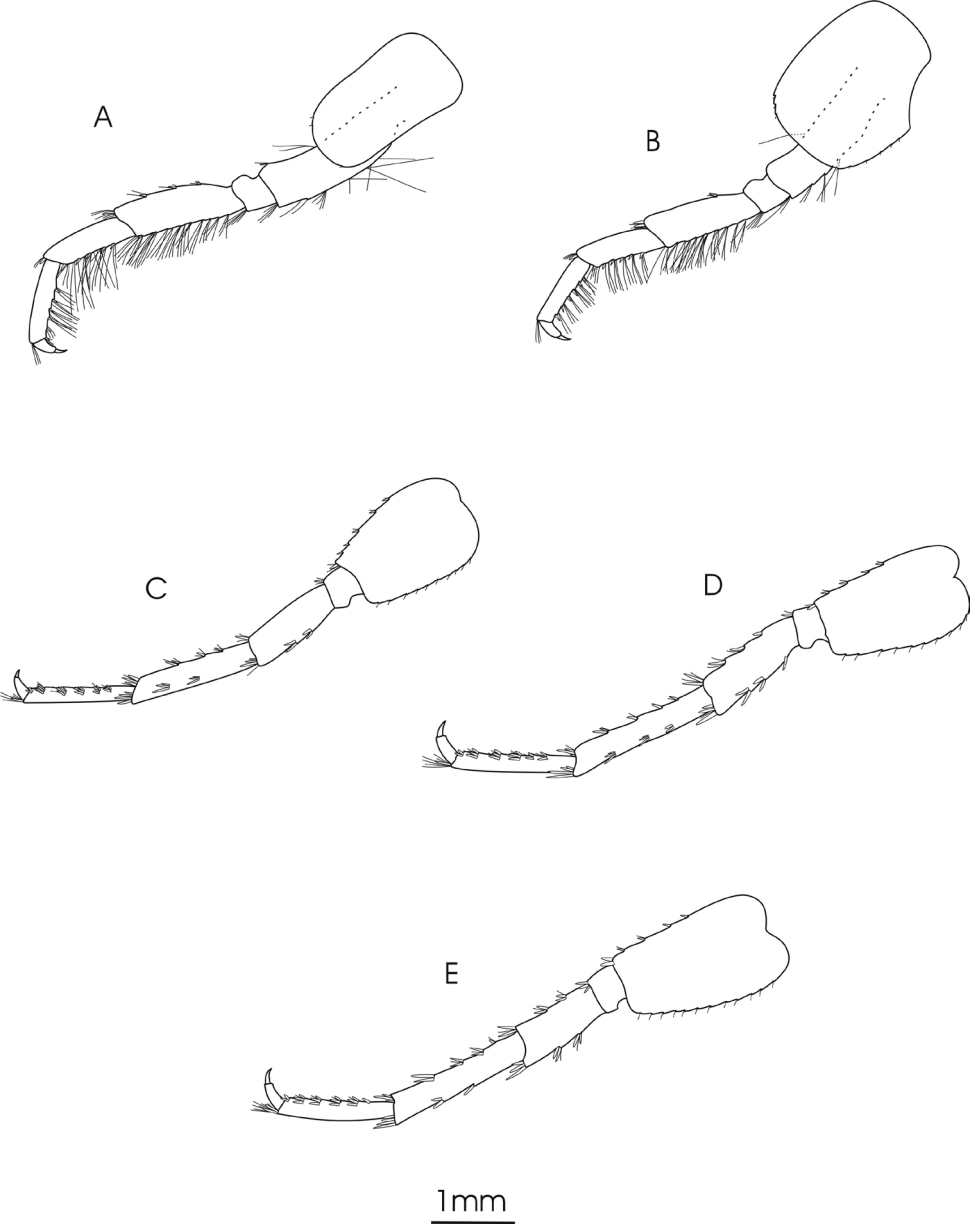
*Gammarus pseudosyriacus issatisi* subsp. n., ♂, 16.9 mm. **A** pereopod 3 **B** pereopod 4 **C** pereopod 5 **D** pereopod 6 **E** pereopod 7.

*Gnathopod 1*: Coxal plate distally slightly wider than proximal, rounded corners with a seta at the postero-ventral corner and 1–3 setae at antero-ventral corner; basis with a few long setae on both anterior and posterior margins; ischium with a postero-distal row of setae; merus and carpus with groups of short setae which are plumose at posterior margin of carpus; propodus pyriform with groups of spines and setae, 5–6 groups of small spines at posterior palmar margin; dactylus long (Fig. 5D). *Gnathopod 2*: Coxal plate distally slightly narrower than proximal, rounded corners with a seta at the postero-ventral corner and 1–3 setae at antero-ventral corner; basis with a few long setae on both anterior and posterior margins; ischium with a postero-distal row of setae; merus and carpus with groups of short setae which are plumose at posterior margin of carpus; propodus Trapezoid-shaped (subrectangular) with 2–3 groups of spines and also groups of dense setae on palmar surface (Fig. 5E). *Pereopod 3*: Coxal plate rectangular and rounded distally, with 2–3 very short setae at antero-distal corner and one at postero-distal corner; anterior and posterior margins of basis bear some long simple setae; posterior margins of merus and carpus densely setose; posterior margin of merus with several groups of dense setae about 1 to 1.5 times as long as the diameter of the article and anterior margin with 2–3 groups of short spine mixed with short setae and a group of long setae with a spine at anterior tip, mean ratio of merus length/width 3.1; posterior margin of carpus with several groups of setae about 2 times longer than the diameter of the article, a long spine and a group of longer setae are implanted on both its anterior and posterior tip, mean ratio of carpus length/width 3.3; posterior margin of propodus with 6 groups of small spine and some long setae (Fig. 7A). *Pereopod 4*: Coxal plate with 2–3 small setae implanted at antero-distal margin and 6–7 at postero-distal margin; articles similar to pereopod 3, but setae are shorter and the number of setae and groups is lower; mean ratio of basis length/width 3.2; anterior margin of merus with just one group of short setae and one spine, two long spines among a group of setae implanted at anterior tip of merus, mean ratio of merus length/width is 2.9; posterior margin of carpus with several groups of setae and spines; posterior margin of propodus with 5–6 groups of one small spine and some long setae (Fig. 7B). *Pereopod 5*: Basis subrectangular, postero-distal lobe well developed, posterior margin with 10–11 very short setae, anterior margin with 4–5 spines; merus and carpus with small spines and setae, mean ratio of merus length/width 2.5; mean ratio of carpus length/width 5.4; propodus having 6 transverse rows of spines (Fig. 7C). *Pereopod 6*: Longer than pereopod 5; basis slender and posterior margin with 10–11 setae and anterior margin with 4–5 spines, mean ratio of basis length/width 1.8; other articles are similar to pereopod 5; mean ratio of merus length/width 2.8; mean ratio of carpus length/width 6.1 (Fig. 7D). *Pereopod 7*: Basis wider proximally, postero-distal protruding lobe less developed than pereopod 6, posterior margin with 11–16 setae and anterior margin with 4–5 spines, mean ratio of basis length/width 1.9; anterior margin of merus and carpus with spines and longer setae; merus with two spine and some short setae at posterior margin; carpus with 1–3 spines at posterior margin; propodus with spines and setae which are as long as spines, 6–7 transverse rows of spines on anterior margin of propodus, two longer spines at posterior tip of propodus intermixed with a group of longer setae (Fig. 7E). *Uropod 3*: Endopodite length is about two-thirds of the exopodite; setae on outer and inner margin of both exopodite and endopodite are plumose; mean ratio of exopodite length/width 5.7 (Fig. 6H).

*Telson*: Length of the lobes about twice their widest width; two to three long and robust spines and 5–6 long setae on distal margin; groups of setae at the dorsal surface of the lobes; mean ratio of telson lobe length/width 1.7 (Fig. 6F). *Epimeral plate 1*: Rounded with 9–12 long setae on antero-distal corner (Fig. 5F). *Epimeral plate 2*: Posterodistal corner pointed; distal margin with 1–4 short spines that mixed with setae (Fig. 5G). *Epimeral plate 3*: Posterodistal corner sharply pointed; 2–4 short spines intermixed with short setae on distal margin (Fig. 5H). *Urosomites 1–3*: With clear dorsal elevation; each urosomite bears a dorsomedian and dorsolateral groups of setae, mixed with short spines on their posterior margins (Fig. 6G).

#### Etymology.

The specific name *issatisi* is an adjective that comes from “Issatis”, which was the previous name of Yazd during the time of the Median Empire. Yazd is an ancient city with a 3,000 year history. The type locality is in the vicinity of Yazd city.

#### Distribution.

*Gammarus pseudosyriacus issatisi* subsp. n. is distributed in several springs and qanats in Yazd Province, in the south of Iran.

#### Ecology.

Qanat–e–Hojjat Abad showed pebbles and a sandy bed. Ecological factors include salinity (0.4 g/lit), pH (7.7), electrical conductivity (733.3 μS/cm), water temperature (13 °C) and water depth (less than 20 cm).

## Discussion

The first record of *Gammarus pseudosyriacus* from Iran (Charmahal-Va-Bakhteyari province) was reported by Khalaji-Pirbalouti and Sari in 2004. In addition, this species was found in other provinces including Markazi Province and Isfahan Province (Naghib 2002) and Kerman Province (Pourmohammadi-Sarbanani 2002) in the far margin of southern Zagros Mountains. These studies show distribution of this species along the Zagros Mountains from the northwest to the southeast (Khalaji-Pirbalouty and Sari 2004, Zamanpoore et al. 2011, Ebrahimnezhad et al. 2005).

A morphological redescription and complete illustrations of *Gammarus pseudosyriacus* are presented. This species shows a high morphological variation across its distribution range (Khalaji-Pirbalouty and Sari 2004, Zamanpoore et al. 2011, Özbek 2011). The original description of this species was presented in an extensive volume (Karaman and Pinkster 1977) describing a large number of new species, so that, as the authors emphasized, “… it was not possible to illustrate all morphological details of every taxon mentioned” (Karaman and Pinkster 1977, p.1), including *Gammarus pseudosyriacus*. For better evaluation of this species for future taxonomic studies, a description of this species in greater detail was prepared.

All major body parts were described, as well as those which were not previously described. These include mandible, maxilla 1(left and right), maxillipeds, first and second gnathopods, and third and fourth pereopods. Complete illustrations are provided, including antenna 1, mandible, maxilla 1 (left and right), maxillipeds, first and second gnathopods, third, fourth and sixth pereopods which were not present in the original paper (except for propodus of gnathopod 1 and 2).

In addition, the Yazd population is introduced as a new subspecies of *Gammarus pseudosyriacus* because of its obvious differences such as smaller eyes, shorter body length, and shorter flagellum of antenna 1 and 2 from the originally described species which we hereby refer to as *Gammarus pseudosyriacus pseudosyriacus*. According to the data from morphometrical study, this subspecies has significant morphological differences from *Gammarus pseudosyriacus pseudosyriacus* in one or several parts of its body organs. It must be noted that there were no seasonal variations in any body parts of the Eghlid population, so it is concluded that these differences are not related to seasonal morphological changes.

In the Zagros Mountains there are many aquatic habitats that were occupied by different populations of *Gammarus pseudosyriacus* and it seems that these populations inhabiting the inner parts of the Zagros are temporally or permanently connected to each other. Eghlid population is one of these Zagros populations which is consistent with the first descriptions of holotype (Karaman and Pinkster 1977), and is therefore considered as a source population. Considering that the source populations have the best and most fit ecological situations, usually the main phenotypes of each species are found in these populations (Mayr 1970). It is suggested that over time the sub-populations of the source population dispersed through the common methods and occupied peripheral habitats. Yazd Province lies in a hot and dry desert, with very few sources of running water, and no existing connection to surface waters of Eghlid and the rest of the Zagros. So the Yazd population can be considered as a sink population. The two populations are established in 2 different catchment basins surrounded by mountains and hills. On the other hand, populations of *Gammarus pseudosyriacus* in Eghlid and Yazd are surrounded and separated by desert plains. These highlands and vast deserts between two habitats have acted as strong geographical barriers which led to long disconnection between two populations and decrease of gene flow.

In terms of ecological characteristics, there are also significant differences between two habitats, including the salinity and electrical conductivity which were much higher in Yazd station (twice). In addition, morphological divergence could have increased as a result of environmental pressures acting in different ways. These factors, along with genetic drift (and even the founder effect) may have led to the formation of the new characters independently. Therefore, it can be concluded that these morphological and probably genetic differences have occurred after separating from the main population.

There are records of isolated populations which are considered as subspecies in different species of amphipods. Cole (1970) described a new subspecies *Gammarus minus pinicollis*, this subspecies in some features such as lack of calceoli, dorso-lateral armature of the urosomites and the ratio of endopod-exopod (exceed 0.67 commonly) differs from *Gammarus minus* Say, 1818. Sutcliffe (2010) introduced two subspecies, *Gammarus duebeni duebeni* and *Gammarus duebeni celticus* based on differences in ratios of merus width to length of pereopod 7. Özbek and Rasouli (2014) described a new subspecies, *Gammarus komareki aznavensis*, that has some features that are different from *Gammarus komareki* Schäferna, 1922 including shorter flagellum of antenna 1, kidney-shaped and bigger eyes, more setose outer margin of the exopodite of uropod 3 and shorter endopodite of uropod 3. Based on our findings on the differences described in the present article, we propose Yazd population to be recognized as a new subspecies, *Gammarus pseudosyriacus issatisi* subsp. n.

## Conclusions

Based on previous studies, *Gammarus pseudosyriacus* is distributed from the northwest to the southeast of the Zagros Mountains. In this study we considered Eghlid population in the inner parts of the Zagros Mountains as a source population. It is connected to other populations of the species, so that it shows no clear variation with the first descriptions of holotype (Karaman and Pinkster 1977). Hereby, we assume it as *Gammarus pseudosyriacus pseudosyriacus* subsp. n. Based on our morphological and morphometric study, revealing significant variations (such as smaller eyes, shorter body length, and shorter flagellum of antenna 1 and 2) between Yazd population and the rest of the populations of *Gammarus pseudosyriacus*, we introduced Yazd population as a new subspecies *Gammarus pseudosyriacus issatisi* subsp. n.

## Supplementary Material

XML Treatment for Gammarus pseudosyriacus pseudosyriacus

XML Treatment for Gammarus pseudosyriacus issatisi

## References

[B1] Cole GA (1970) *Gammarus minus*: geographic variation and description of new subspecies *G. m. pinicollis* (Crustacea, Amphipoda). Transactions of the American Microscopical Society, 514–523. doi: 10.2307/3224561

[B2] Ebrahimnezhad M Hosseini L Sari A (2005) Collecting and identification of *Gammarus* species of the Zayandeh-rood River. Majalle ye Zistshenasi-e Iran 18(3): 218–227.

[B3] Fabricius JC (1775) Systema Entomologiae, sistens insectorum classes, ordines, genera, species, adiectis synonymis, locis, descriptionibus, observationibus. Officina Libraria Kortii, Flensburgi et Lipsiae, 832 pp.

[B4] Karaman GS Pinkster S (1977) Freshwater *Gammarus* species from Europe, North Africa and adjacent regions of Asia (Crustacea, Amphipoda), part 1.*Gammarus pulex*-group and related species. Bijdragen Tot De Dierkunde 47: 1–97.

[B5] Khalaji-Pirbalouty V Sari A (2004) Biogeography of amphipods (Crustacea: Amphipoda: Gammaridae) from the central Zagros Mountains, Iran, with descriptions of two new species. Journal of Natural History 38(19): 2425–2445. doi: 10.1080/00222930310001647406

[B6] Mayr E (1970) Populations, species, and evolution: an abridgment of animal species and evolution. Harvard University Press, Harvard, 453 pp.

[B7] Naghib M (2002) A study on distribution, embryology and karyology of amphipods from Qom and Isfahan provinces. MSc Thesis, University of Tehran, Tehran, Iran.

[B8] Oshel PE Steele DH (1988) Comparative morphology of amphipod setae, and a proposed classification of setal types. Crustaceana Supplement 13: 90–99.

[B9] Özbek M (2011) An overview of the *Gammarus* Fabricius (Gammaridae: Amphipoda) species of Turkey, with an updated checklist. Zoology in the Middle East 53(1): 71–78. doi: 10.1080/09397140.2011.10648863

[B10] Özbek M Rasouli H (2014) *Gammarus komareki aznavensis* subsp. nov., a new amphipod subspecies from Iran (Amphipoda: Gammaridae). Turkish Journal of Zoology 38(3): 326–333. doi: 10.3906/zoo-1306-1

[B11] Pourmohammadi-Sarbanani M (2002) A study on species and populations of Amphipoda (Crustacea) in Kerman province with emphasis on aquaculture. MSc Thesis, University of Tehran, Tehran, Iran.

[B12] Schäferna K (1922) Amphipoda Balcanica. Vëstnik Kral. C. Spoleinosti Nauk Ti-ida mat.-prir. Praha, 2, 1–110.

[B13] Sutcliffe D (2010) Subspecies, morphs and clines in the amphipod *Gammarus duebeni* from fresh and saline waters. Freshwater Forum 13: 60–75. http://aquaticcommons.org/id/eprint/4602

[B14] Väinölä R Witt JDS Grabowski M Bradbury JH Jazdzewski K Sket B (2008) Global diversity of amphipods (Amphipoda; Crustacea) in freshwater. Hydrobiologia 595: 241–255. doi: 10.1007/s10750-007-9020-6

[B15] Zamanpoore M Grabowski M Poeckl M Schiemer F (2010) Two new *Gammarus* species (Crustacea, Amphipoda) from warm springs in the south-east pre-alpine area of the Zagros, Iran: habitats with physiological challenges. Zootaxa 2546: 31–51.

[B16] Zamanpoore M Poeckl M Grabowski M Schiemer F (2011) Taxonomic review of freshwater *Gammarus* from Iran. Zootaxa 3140: 1–14.

